# Bovine and Ovine Teeth as a Substitute for the Human Teeth: An Experimental Study

**DOI:** 10.30476/dentjods.2022.94500.1792

**Published:** 2024-06-01

**Authors:** Safoura Sahebi, Fereshte Sobhnamayan, Soheila Hasani, Negar Mahmoodi, Delara Dadgar

**Affiliations:** 1 Dept. of Endodontics, School of Dentistry, Shiraz University of Medical Sciences, Shiraz, Iran; 2 Postgraduate Student, Dept. of Endodontics, School of Dentistry, Shiraz University of Medical Sciences, Shiraz, Iran; 3 Undergraduate Student, Dept. of Endodontics, School of Dentistry, Shiraz University of Medical Sciences, Shiraz, Iran; 4 Postgraduate Student, Dept. of Clinical Pathology, Faculty of Specialized Veterinary Sciences, Islamic Azad University, Science and Research Branch, Tehran, Iran

**Keywords:** Dentin, Calcium hydroxide, Tooth Roots

## Abstract

**Statement of the Problem:**

Although various kinds of research have been conducted to compare the physical and chemical properties of dentin and enamel in animal and human samples, proving the ability of animal dentin material as a good substitute for human specimens is always a challenge for experimental studies.

**Purpose:**

The aim of the present study is to investigate whether the changes in the dentin microhardness of animal samples are similar to those of human samples or not.

**Materials and Method:**

In this *in vitro* study, sixty single-rooted human, bovine, and ovine teeth (n=20 in each group) were decoronated at CEJ. The remaining roots were embedded in acrylic resin and a cross-section cut was made in the middle of the samples in order to achieve dentin disks. All of the 120 samples were randomly assigned to three control (n=20 for each group) and three experimental groups (n=20 for each group). In the experimental groups, calcium hydroxide with a creamy consistency was prepared and the disks were embedded in dishes containing calcium hydroxide. Control groups were embedded in physiological saline. The samples were incubated for seven days at the 37oC and Vickers microhardness test was performed immediately. The average of three yielded values was considered as the final value of microhardness. Data were analyzed using two-way ANOVA, one-way ANOVA, and Tukey’s post hoc tests.

**Results:**

In the control group, the human samples showed the highest microhardness value, while the bovine teeth had the lowest microhardness value (*p*< 0.001). In the calcium hydroxide group, the human samples showed the highest microhardness value in comparison to bovine and ovine to teeth. However, no significant difference was observed between the bovine and ovine samples in microhardness value.

**Conclusion:**

Based on our research, substituting bovine and ovine samples with human samples in experimental studies is not recommended. Nevertheless, more studies are needed in this regard.

## Introduction

Animal samples are used for *in vitro* studies since there are limitations in accessing human samples. Samples obtained from human teeth are preferred for dental research as they could simulate the clinical conditions. However, the difficulty in obtaining human teeth with acceptable quality, ethical issues, and infection hazards have prompted researchers to substitute human samples with animal samples [ 1 - 3 ].

Several types of animal teeth have been used as a substrate for chemical and physical dental experiments. Bovine, ovine, equine, porcine teeth are some examples in this regard [ 4 , 6 - 8 ]. The most important criteria for choosing animal teeth as a substitute for human teeth are the similarity of their physiochemical structures and biological characteristics to those of human teeth [ 7 ]. Nevertheless, none of these animal samples has the same characteristics as human samples. Over the past 30 years, bovine teeth have been frequently used as a substitute for human teeth as their collection is easy [ 9 ]. Although standardizing animal samples based on tooth age, diet, and environmental conditions is very difficult, it could reduce bias in dental experiments [ 9 ]. Some studies [ 5 - 7 ] have compared bovine dentin and human dentin. They have reported that bovine dentin can be an appropriate substitute for human teeth. Castanho *et al*. [ 10 ] showed that both human and bovine sclerotic dentin have a similar amount of solid tissue although human sclerotic dentin has a higher microhardness than bovine sclerotic dentin. Teruel *et al*. [ 11 ] also reported that bovine dentin and enamel had a higher similarity to human teeth than porcine and ovine teeth. In another research, Tanaka *et al*. [ 12 ] demonstrated that the radiographic density of bovine radicular dentin was lower than that of human radicular dentin although this difference was not significant.

Due to contradictory data in different papers, it has been suggested that the differences in the morphological, chemical, and physical characteristics between human and bovine teeth be considered in interpreting the results. Very few studies have used ovine teeth as an alternative to human teeth. The main reason for the studies in this regard was that the root anatomy of these teeth was comparable to that of human incisors [ 13 ]. Dehghani Nazhvani *et al*. [ 7 ] showed that from ultrastructural and chemical viewpoints, the enamel and dentin of sheep and guinea pigs had the least resemblance to human ones, while the enamel and dentin of dogs and cats had the highest resemblance to human ones.

Besides their advantages, different irrigants and medicaments in endodontic treatment might have detrimental effects on the mechanical properties of dentin [ 14 ]. Among these, calcium hydroxide paste is widely used in endodontic treatments [ 15 - 17 ] due to its antibacterial effects [ 18 - 20 ], tissue-dissolving properties [ 21 - 22 ], and ability to induce hard tissue barrier formation [ 23 - 24 ]. It has also been utilized in vital pulp therapy [ 23 ], apexification procedures [ 25 ], and inflammatory root resorption [ 26 ].

Yoldas *et al*. [ 27 ] reported a significant reduction in dentin microhardness following the use of calcium hydroxide combinations after 3 and 7 days of exposure. Naseri *et al*. [ 28 ] also showed that the application of calcium hydroxide in human dentin significantly reduced its microhardness, whereas the nano-calcium hydroxide did not result in any change in the microhardness value. Zareie *et al*. [ 29 ] indicated that the fracture resistance of human root dentin exposed to calcium hydroxide for one week did not change significantly compared with that of the control group. Inconsistent with previous studies, Kawamoto *et al*. [ 30 ] reported that exposure to calcium hydroxide paste increased the elastic modulus of dentin and made it more susceptible to fracture [ 30 ]. 

Although various researches have been conducted to compare the physical and chemical properties of dentin and enamel in animal and human samples, proving the ability of animal specimens as a good substitute for human specimens has always been a challenge. No studies have been conducted to show whether the behavioral changes of animal samples in the presence of materials such as calcium hydroxide are similar to those of human samples or not. The aim of the present study was to investigate whether the changes in the dentin microhardness of animal samples were similar to those of human samples or not.

## Materials and Method

**Figure 1:**
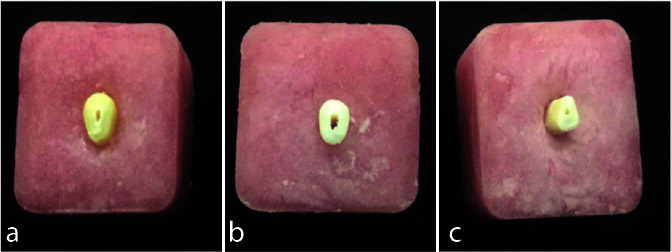
**a:** Human sample, **b:** Bovine sample, **c:** Ovine sample

Twenty intact single-rooted permanent human teeth extracted for orthodontic reasons were collected. The patients’ informed consent and the approval of the Ethics in Human Research Committee of Shiraz University of Medical Sciences were obtained. This *in vitro* study was approved by the Ethics Committee of Shiraz University of Medical Science (IR.SUMS.DENTAL.REC. 1399.034). Bovine and ovine mandible incisors (20 of each) with similar sizes and shapes and without cracks were selected. The teeth were cleaned and stored in 0.4% thymol solution until the beginning of the experiment. The teeth were decoronated at the CEJ level with a water-cooled diamond saw (Microdont, LDA, Brazil) at 1000 rpm. The remaining roots were embedded in an acrylic self-cure resin (Acrostone, Dent Product, Egypt). Then, a cross-section cut was made in the middle of each specimen using a low-speed and water-cooled rotating diamond disk so that the dentin thickness around the canal of each specimen was about 3mm. Ultimately, the human, bovine, and ovine samples were obtained. The samples were polished using water-cooled 500, 800, 1000, and 1200 grit abrasive papers (Bigo, Dent Product, Germany), a felt disk (Microdent, LDA, Brazil), and a 0.1ml alumina suspension (Figure 1).

All of the 120 samples were randomly assigned to three control (n=20 for each group) and three experimental groups (n=20 for each group). In the control group, human, bovine, and ovine samples (n=20 in each group) were embedded in Petri dishes containing physiological saline. In the experimental groups, calcium hydroxide (Merck, Darmstadt, Germany) was prepared with a creamy consistency (powder to saline ratio: 3/1) and the disks were embedded in dishes containing calcium hydroxide. 

During the 7 days of the experiment, the samples were incubated at the temperature of 37 ℃ and moisture of 100%. At the end of this period, the samples were rinsed with physiological saline and Vickers microhardness test was performed immediately. Three indentations were made on the dentin surface of each cylinder at the distance of 1mm from the root canal space. A 500-gr load was applied to the samples for 10 seconds. The average of the three obtained numbers was considered as the final value of microhardness [ 5 ]. The data were analyzed using two-way ANOVA, one-way ANOVA, and Tukey’s post-hoc tests. Data collected and statistically analyzed with IBM^®^ SPSS^®^ Statistics Version 25 (SPSS Inc., IBM Corporation, NY, USA).

## Results

**Figure 2:**
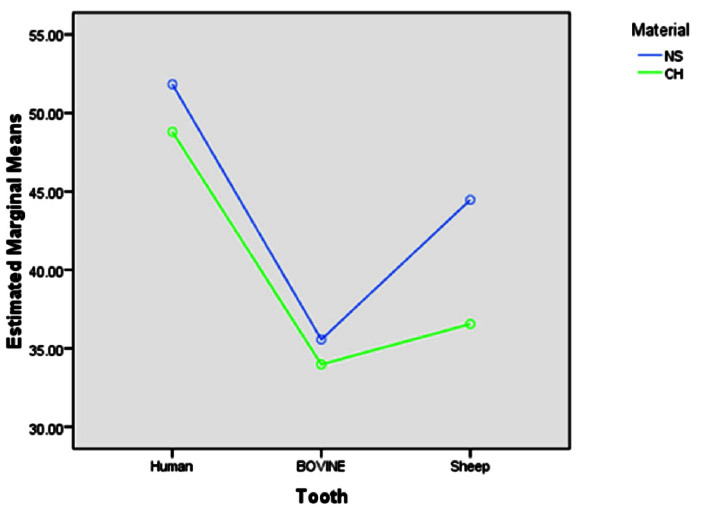
Evaluation of interaction effect of normal saline (NS), and calcium hydroxide (CH)

The two-way ANOVA test demonstrated an interaction effect between different teeth groups (human, bovine, and ovine) (Figure 2). Table 1 shows the mean value of microhardness in each group.

**Table 1:** comparison of the microhardness in all experimental groups after 7-day medicament exposure

Variables	Human	Bovine	Ovine	*p* ^**^
Normal Saline	51.83±2.55^a^	35.57±2.97^b^	44.47±5.21^c^	<0.001
Calcium Hydroxide	48.80±.96^a^	35.98±2.42^b^	36.56±2.49^c^	<0.062
p**	0.019	0.072	<0.001	

p^*^: independent T test

p^**^: one way ANOVA F test

In each column mean values with different letters were statistically significant (Tukey’s post-hoc test)

The results showed that using calcium hydroxide significantly decreased the microhardness of human and ovine dentin (*p*= 0.019 and *p*< 0.0001, respectively), although it was not significant for bovine teeth (*p*= 0.072).

In the control group, the microhardness values of the human and animal samples were significantly different from each other so that the human teeth had the greatest microhardness value and the bovine teeth showed the lowest microhardness value (*p*< 0.001).

In the calcium hydroxide groups, the microhardness value of the human samples was significantly higher than that of bovine and ovine samples. However, no significant difference was observed between the microhardness values of bovine and ovine samples (*p*= 0.062).

## Discussion

The purpose of this research was to examine the possible deleterious effects of calcium hydroxide on human bovine and ovine root dentin and to compare these dentin structures to each other in order to determine if bovine and ovine teeth could be appropriate substitutes for human dentin.

The present study showed that the microhardness value of human dentin was significantly higher than that of bovine and ovine teeth, both in the control and calcium hydroxide groups. The microhardness value of ovine teeth was significantly higher than that of bovine teeth in the control groups. The higher microhardness value of human dentin compared to bovine dentin has been confirmed by previous studies [ 10 , 31 - 32 ]. Teruel *et al*. [ 11 ] and Cochrane *et al*. [ 32 ] both reported that the microhardness value of human dentin was higher than that of bovine dentin. Another study also showed that human radicular dentin was more radiodense than bovine radicular dentin [ 12 ]. This phenomenon and the higher microhardness value of human samples compared to bovine ones could be explained by the higher degree of mineralization in human dentin. Thus, Castanho *et al*. [ 10 ] suggested using bovine sclerotic dentin instead of human sclerotic dentin because they have a similar amount of solid tissue. 

The present study showed that the dentin microhardness of ovine teeth was more similar to the dentin microhardness of human teeth than that of bovine teeth. In their studies, Dehghani Nazhvani *et al*. [ 7 ] and Oritz-Ruiz *et al*. [ 6 ] showed that there were differences in the organic and inorganic contents of human and other animal samples. From the ultrastructural and chemical aspects, Dehghani Nazhvani *et al*. [ 7 ] reported that dog human dentin and enamel had the greatest resemblance to human ones and sheep and guinea pigs had the least resemblance to human ones.

Teruel *et al*. [ 11 ] also reported the greater microhardness value of human dentin compared to ovine dentin, which is due to the higher degree of mineralization in human dentin. Although Altai *et al*. [ 33 ] suggested ovine samples as a good substitute for human samples in revascularization procedures, this suggestion arose from the similarity of these samples in their dimensions and anatomic characteristics rather than the similarity of their compositions as well as physical and chemical characteristics. The contradictory results in different studies could be attributed to the differences in the age and diet of the animal samples as well as the different kinds of chemical and physical tests, which were performed.

In agreement with the present study, several studies have shown that the short-term use of calcium hydroxide reduces the microhardness of dentin and even increases the elastic modulus of dentin, making it more susceptible to fracture [ 22 , 27 , 30 ]. On the other hand, some other studies have shown that the short-term use of calcium hydroxide for seven days [ 28 - 29 , 34 ] or thirty days [ 33 ] does not have an adverse effect on the microhardness of dentin. These contradictory results could be attributed to the different types and concentrations of calcium hydroxide used in these studies, different tests (Knoop or Vickers test) used to evaluate the microhardness, different preparations of dentin samples, and even the age of the teeth. All these factors could greatly affect the results. Other studies which evaluated the effect of calcium hydroxide on animal samples also showed contradictory results [ 13 , 30 , 34 - 35 ]. 

Many studies have used animal samples as a substitute for human samples to evaluate the effects of different irrigants and medicaments on the mechanical properties of dentin [ 5 , 13 , 30 ]. The current study was designed to evaluate the effects of calcium hydroxide on the microhardness of bovine, ovine, and human samples to determine whether these animal samples showed the same characteristics and changes as human samples in the presence of calcium hydroxide. There are significant differences between the anatomical, physical, and chemical properties of human and animal dentin samples [ 7 ]. In addition to these differences, this study emphasized the behavioral changes of these samples in the presence of calcium hydroxide. Many studies [ 5 , 27 , 29 - 30 ] have confirmed that calcium hydroxide, by its nature, could reduce the microhardness of dentin. The results of the present study showed the different behaviors of animal dentin samples compared with the human ones in the presence of calcium hydroxide. The more reduction in the microhardness values of ovine and bovine dentin in the presence of calcium hydroxide compared to human dentin samples does not make animal samples good candidates for substitution in microhardness studies. 

On the other hand, Kahler *et al*. [ 25 ] and Hawkins *et al*. [ 36 ] suggested that calcium hydroxide was not associated with a reduction in the fracture resistance of ovine teeth. Instead, the thin and fragile roots of ovine teeth in different developmental stages could be the cause of fracture. These contradictory results arise from the differences in the tests performed in different studies.

Some studies have evaluated flexural strength [ 5 ], some have investigated the modulus of elasticity [ 30 ], and some have tested microhardness [ 27 ]. Even the type of microhardness test is different in each paper. The differences in the concentration of calcium hydroxide paste, the number of experiment days, and other variables in these studies have led to these differences. 

## Conclusion

According to the results of the present study and regarding the contradictory results of other studies, it seems that bovine and ovine teeth could not be considered as good substitutes for human teeth unless human samples are not available. On the other hand, the short-term use of calcium hydroxide could have a detrimental effect on human and ovine teeth; therefore, it should be applied cautiously. Further testing is required to examine the other mechanical properties of dentin samples as well as determining the clinical relevance of these findings.
